# Lipid-mediated antimicrobial resistance: a phantom menace or a new hope?

**DOI:** 10.1007/s12551-021-00912-8

**Published:** 2022-02-25

**Authors:** Hugo I. MacDermott-Opeskin, Vrinda Gupta, Megan L. O’Mara

**Affiliations:** Research School of Chemistry, College of Science, The Australian National University, Canberra, ACT 2601 Australia

**Keywords:** Bacterial lipids, Antimicrobial resistance, Lipidomics, Bacterial membranes, Antimicrobial peptides, Molecular dynamics simulation, Experimental characterisation

## Abstract

The proposition of a post-antimicrobial era is all the more realistic with the continued rise of antimicrobial resistance. The development of new antimicrobials is failing to counter the ever-increasing rates of bacterial antimicrobial resistance. This necessitates novel antimicrobials and drug targets. The bacterial cell membrane is an essential and highly conserved cellular component in bacteria and acts as the primary barrier for entry of antimicrobials into the cell. Although previously under-exploited as an antimicrobial target, the bacterial cell membrane is attractive for the development of novel antimicrobials due to its importance in pathogen viability. Bacterial cell membranes are diverse assemblies of macromolecules built around a central lipid bilayer core. This lipid bilayer governs the overall membrane biophysical properties and function of its membrane-embedded proteins. This mini-review will outline the mechanisms by which the bacterial membrane causes and controls resistance, with a focus on alterations in the membrane lipid composition, chemical modification of constituent lipids, and the efflux of antimicrobials by membrane-embedded efflux systems. Thorough insight into the interplay between membrane-active antimicrobials and lipid-mediated resistance is needed to enable the rational development of new antimicrobials. In particular, the union of computational approaches and experimental techniques for the development of innovative and efficacious membrane-active antimicrobials is explored.

## Introduction

Antimicrobial resistance (AMR) is one of the foremost threats facing global public health. A 2016 review on antimicrobial resistance gave a conservative estimate of 700,000 deaths caused by AMR annually (O’Neil Jim 2016). As a result of the continuing rise of AMR infections in conjunction with limited advances in the development of novel antimicrobials, the number of AMR-related deaths is predicted to increase to an alarming 10 million annually by 2050 (O’Neil Jim 2016). Traditional antimicrobial therapeutics generally function either by altering bacterial cell wall synthesis (β-lactams, glycopeptides), inhibiting protein or nucleic acid synthesis (macrolides, quinolones and tetracyclines), or interfering with metabolic pathways (sulfonamides) (Reygaert 2018; Streicher 2021). Treatment strategies often use a combination of antimicrobials to simultaneously target multiple biochemical sites. Resistance to a single antimicrobial can arise through a myriad of point mutations in different genes, whilst resistance to combination therapies commonly occurs when several mutations are acquired which reduce or eliminate susceptibility to multiple antimicrobials, making the pathogen multidrug resistant (MDR) (Alekshun and Levy 2007). The ongoing acquisition of AMR against most currently available antimicrobials requires new and innovative solutions. The bacterial cell membrane is the primary barrier for antimicrobial entry to the cell and is a critical mediator of antimicrobial resistance and pathogen survival. As such, the bacterial cell membrane is an attractive target for the development of new antimicrobials (Moellering 2011; Hurdle et al. 2011; Mingeot-Leclercq and Décout 2016; Mehta et al. 2021).

In this mini-review, we outline the lipid-mediated mechanisms by which the bacterial cell membrane causes and controls resistance, particularly focusing on alterations of membrane lipid composition, the chemical modification of membrane lipids and the role of multidrug efflux systems. We also highlight the need to gain a comprehensive understanding of the interplay between lipid-mediated AMR and the mechanisms of antimicrobial action and efflux to guide the rational development of new and effective membrane-active antimicrobials.

### The bacterial cell membrane and membrane-active antimicrobials

Bacterial membranes are dynamic and heterogenous assemblies of macromolecules that contain lipids, proteins and glycans, and act as the primary physical barrier for the cell entry of antimicrobial agents (Strahl and Errington 2017; May and Grabowicz 2018; Willdigg and Helmann 2021). Consequently, membrane-active antimicrobial agents which target the bacterial membrane, either by disrupting the functional integrity of the bacterial membrane itself or modulating the function of essential membrane-associated proteins, can greatly hinder bacterial viability (Zasloff 2002; Fjell et al. 2012; Mingeot-Leclercq and Décout 2016). Although there is widespread resistance to many current classes of antimicrobials, the highly conserved and essential nature of the bacterial membrane would suggest a reduced potential for bacteria to acquire resistance to membrane-active antimicrobials (Hurdle et al. 2011; Fjell et al. 2012; Spohn et al. 2019). Furthermore, as the bacterial membrane and its associated components act as the primary barriers for antimicrobial entry, targeting membrane function and integrity can allow for increased sensitivity to other co-administrated antimicrobials (Mingeot-Leclercq and Décout 2016; Pizzolato-Cezar et al. 2019). One such example is the synergy between azithromycin and the membrane-active antimicrobial peptides, LL-37 and colistin in the treatment of several MDR Gram-negative pathogenic bacteria (Lin et al. 2015).

**Fig. 1:**
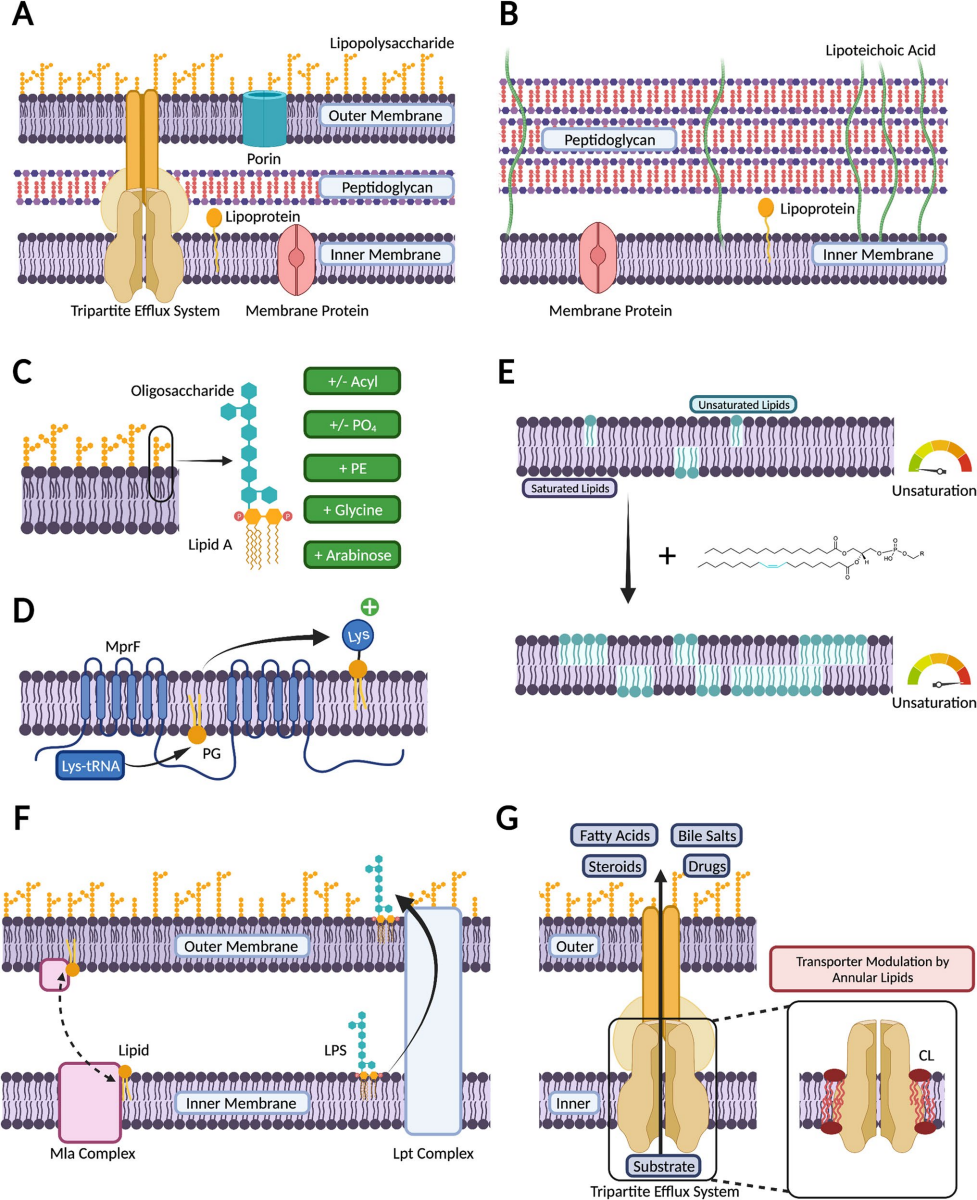
Lipid-mediated mechanisms of AMR. (A) Diagrammatic representation of the Gram-negative bacterial cell envelope and (B) the Gram-positive bacterial cell envelope. (C) Chemical modifications of Lipid A and (D) lysylation of PG provide antimicrobial resistance. (E) Lipid acyl tail remodelling, such as increases in overall membrane unsaturation levels lead to changes in membrane biophysical properties, and control antimicrobial susceptibility. (F) The transport of lipids between membranes and across membrane leaflets by lipid transport systems, including the Mla and Lpt complexes, governs membrane-antimicrobial interactions. (G) Membrane-embedded drug efflux pumps can actively efflux a range of antimicrobials and are regulated by the membrane lipid environment. Figure created with BioRender.com

The different cellular architectures of Gram-positive and Gram-negative bacteria are critical to the functional mechanism of membrane-active antimicrobials (Fig. 1A and B). Gram-negative bacteria contain two membranes; an inner cytoplasmic membrane and an outer membrane, separated by a thin peptidoglycan layer. The inner membrane of Gram-negative bacteria is composed of lipids and integral membrane proteins, whilst the outer membrane also contains lipoproteins, porins and lipopolysaccharides (LPS), found primarily in the extracellular leaflet (Silhavy et al. 2010). In comparison, Gram-positive bacteria possess a single cytoplasmic membrane and a thicker peptidoglycan cell wall. The membrane of Gram-positive bacteria is comprised of phospholipids, integral and associated membrane proteins, and lipoteichoic acid (LTA) components that anchor the membrane to the cell wall (Silhavy et al. 2010). The additional barrier presented by the outer membrane in Gram-negative bacteria makes them a particularly challenging antimicrobial target and has contributed to the rise of numerous strains of MDR Gram-negative pathogens (Brown and Wright 2016). Indeed, nine out of the twelve bacterial pathogens listed by the WHO on their AMR priority list are Gram-negative (WHO 2017).

Within bacterial membranes, the distribution and chemical composition of membrane lipids is highly varied across different species, and even different bacterial strains (Sohlenkamp and Geiger 2015; López-Lara and Geiger 2017). The precise lipid composition of a given membrane has clear implications for the modulation of membrane biophysical properties and the function of membrane-embedded proteins, which in turn governs the activity of membrane-active antimicrobials (Harayama and Riezman 2018; Lee et al. 2019b). Mammalian cell membranes are largely composed of phosphatidylcholine (PC) lipids and cholesterol, whilst bacterial membranes are rich in zwitterionic phosphatidylethanolamine (PE) lipids, anionic phosphatidylglycerol (PG) lipids and poly-anionic cardiolipin (CL). Within these major lipid classes, there is considerable species- and environment-dependent diversity in acyl chain lengths, degree of saturation, and the incorporation of branched chain, cyclopropane-containing, or ω-alicyclic fatty acyl chains (Oshima and Ariga 1975; Sohlenkamp and Geiger 2015; López-Lara and Geiger 2017). Additionally, there are a number of other unique lipid components associated with bacterial membranes. These include endotoxic LPS, composed of Lipid A and the polysaccharide O-antigen; Lipid II, a precursor for bacterial cell wall synthesis; and LTA, which anchors the cell wall of Gram-positive bacteria to the membrane (Epand and Epand 2009a; Silhavy et al. 2010). Notably, the differential distribution of lipid species between bacterial and mammalian membranes, as well as between bacterial species can be used to design membrane-active antimicrobials with highly specific bacterial selectivity (Dias and Rauter 2019).

## Bacterial alterations to membrane lipid composition—the phantom menace

Both Gram-negative and Gram-positive pathogens can alter their membrane composition to adapt to their biological niche (Adams et al. 2021) or evade antimicrobials and host immune mechanisms (Epand and Epand 2009b; Hewelt-Belka et al. 2016; Hines et al. 2017; Sperandeo et al. 2019). These changes in their membranes are induced via transcriptional control of lipid synthesis, or through alterations to the active transport of lipids between membrane leaflets, and also between the inner and outer membranes of Gram-negative bacteria. Consequently, resistant bacterial strains often possess functional mutations in the proteins or regulatory elements controlling membrane composition, lipid synthesis, and lipid transport systems (Hachmann et al. 2009, 2011; Olaitan et al. 2014; May and Grabowicz 2018; Jiang et al. 2019a). As a result, lipid composition-dependent changes in membrane biophysical properties, such as surface charge, membrane thickness, membrane fluidity, and modulation of curvature are heavily implicated in antimicrobial resistance (Epand et al. 2016). In addition, changes in the surface display of signalling or structural lipids (Soto et al. 2019; Giordano et al. 2020), and lipid modifications that change the propensity of the membrane to form segregated domains (Epand and Epand 2009b) have also been highlighted as key determinants of AMR phenotypes.

### Surface charge modification

Membrane surface charge plays a major role in controlling the susceptibility of bacteria to membrane-active antimicrobials (Table 1). As the major lipid species in bacterial membranes are zwitterionic PE, anionic PG and poly-anionic CL, the relative concentration of anionic PG and CL in the bacterial membrane imparts an overall negative surface charge. This provides an electrostatic basis for interactions with charged membrane-active antimicrobials, particularly cationic antimicrobial peptides (CAMPs). Consequently, changes in the relative ratios of these lipid classes alters the overall surface charge of the membrane and governs the strength of membrane interactions with antimicrobial agents. CAMP-resistant bacterial isolates have been observed to carry functional mutations in the pgsA (PG) or cls2 (CL) lipid synthesis enzymes, resulting in a net reduction in the overall negative charge of the membrane surface (Hachmann et al. 2011; Davlieva et al. 2013; Hines et al. 2017; Jiang et al. 2019a). This reduces the propensity of CAMPs to aggregate on the membrane surface, providing resistance against these antimicrobials.

**Table 1:** Summary of bacterial membrane modifications implicated in AMR^a^

Membrane modification	Genetic basis	Mechanism	Bacterial species	Antimicrobial	References
Surface charge modification	*pgsA*	Phosphatidylglycerol synthase	*Bacillus subtilis*	Daptomycin	(Hachmann et al. 2011)
			*Enterococcus faecalis, Staphylococcus aureus, Corynebacterium striatum*		(Hines et al. 2017)
	*cls*	Cardiolipin synthase	*Enterococcus faecium, Enterococcus faecalis*	Daptomycin	(Davlieva et al. 2013)
	*cls2*		*Staphylococcus aureus*		(Jiang et al. 2019a)
Lipid chemical modifications	*lpxE, lpxF*	Lipid A phosphatase	*Rhizobium etli*	Polymyxin B	(Ingram et al. 2010)
	*lpxF*		*Bacteroides thetaiotaomicron*		(Cullen et al. 2015)
	*lpxE*		*Francisella novicida*	﻿Bacitracin	(Zhao et al. 2019)
	*pagP*	Lipid A palmitoyltransferase	*Salmonella typhimurium*	C18G, pGLa, Protegrin, Polymyxin B	(Guo et al. 1998)
	*pagL*	Lipid A deacylase	*Salmonella typhimurium*	Toll-like receptor 4 signalling	(Kawasaki et al. 2004)
	*mcr-1*	Lipid A phosphatidylethanolamine transferase	*Escherichia coli, Klebsiella pneumoniae, Acinetobacter baumannii, Pseudomonas aeruginosa*	Colistin	(Liu et al. 2017)
	*almG*	Lipid A glycyltransferase	*Vibrio cholerae*	Polymyxin B	(Henderson et al. 2017)
	*pmrA, pmrE, pmrF*	Lipid A modification with L-Ara4N and phosphatidylethanolamine	*Salmonella typhimurium*	Polymyxin	(Zhou et al. 2001)
	*mprF*	Phosphatidylglycerol lysyltransferase/flippase	*Staphylococcus aureus*	NK-2, Arenicin-1,	(Andrä et al. 2011)
				Thrombin-induced platelet microbicidal proteins, hNP-1, Polymyxin B	(Mishra et al. 2011)
			*Enterococcus faecalis*	Pediocin, Nisin, Alamethicin	(Kumariya et al. 2015)
			*Staphylococcus aureus*	Daptomycin, Friulimicin B	(Ernst et al. 2018)
				Daptomycin	(Sabat et al. 2018)
	*lpiA*	Involved in lysyl-phosphatidylglycerol synthesis	*Rhizobium tropici*	Polymyxin B	(Sohlenkamp et al. 2007)
	PA0920	Alanyl-phosphatidylglycerol synthase	*Pseudomonas aeruginosa*	Cefsulodin, Protamine	(Klein et al. 2009)
	aaPGSs	Multiple aminoacyl phosphatidylglycerol synthases	*Enterococcus faecium,* *Bacillus subtilis, Clostridium perfringens, Agrobacterium tumefaciens*	N/A	(Roy and Ibba 2009)
	*dltA* *dltB* *dltC* *dltD*	D-alanylation of teichoic acids	*Clostridium difficile*	Nisin, Polymyxin B, Gallidermin, Vancomycin	(McBride and Sonenshein 2011)
	*dltA*		*Staphylococcus aureus*	Daptomycin	(Sabat et al. 2018)
Acyl tail remodelling	N/A	Alterations to membrane fluidity	*Streptococcus pneumoniae*	N/A	(Aricha et al. 2004)
	N/A		*Enterococcus faecalis*	Pediocin, Nisin, Alamethicin	(Kumariya et al. 2015)
	N/A	Incorporation of exogenous fatty acids	*Enterococcus faecalis*	Daptomycin	(Saito et al. 2014)
	N/A				(Harp et al. 2016)
	N/A	Alterations to membrane homeostasis	*Acinetobacter baumannii*	Colistin	(Tao et al. 2021)
	N/A	Alterations to membrane fluidity	*Escherichia coli, Staphylococcus aureus*	Naringenin	(Wang et al. 2018)
	*cfa*	Cyclopropane-fatty-acyl-phospholipid synthase	*Escherichia coli*	Apidaecin 1b	(Schmidt et al. 2018)
	N/A	Alterations to membrane homeostasis	*Enterococcus faecalis, Staphylococcus aureus, Corynebacterium striatum*	Daptomycin	(Hines et al. 2017)
	*fadL*	Incorporation of exogenous fatty acids	*Acinetobacter baumannii*	N/A	(Adams et al. 2021)
Lipid domains and curvature	N/A	Targeting cardiolipin domains	*Pseudomonas aeruginosa*	3’,6-dinonyl neamine	(El Khoury et al. 2017)
					(Swain et al. 2018)
	N/A	Increased glycolipid content	*Enterococcus faecalis, Staphylococcus aureus, Corynebacterium striatum*	Daptomycin	(Hines et al. 2017)
	N/A	Alterations of membrane domains and protein localisation	*Bacillus subtilis*	cWFW	(Scheinpflug et al. 2017)

^a^Key references from this review are presented, and entries are ordered as they appear in text

### Lipid chemical modifications

Another mechanism bacteria employ to reduce membrane surface charge is the chemical modification of constituent lipids (Fig. 1C and D, Table 1). Gram-negative pathogens readily modify Lipid A through a wide variety of enzymatic mechanisms, including dephosphorylation (Ingram et al. 2010; Cullen et al. 2015; Zhao et al. 2019), acylation (Guo et al. 1998), diacylation (Kawasaki et al. 2004), addition of various sugars and decoration with PE (Liu et al. 2017), glycine (Henderson et al. 2017), or aminoarabinose (Zhou et al. 2001; Raetz et al. 2007; Olaitan et al. 2014; Sperandeo et al. 2019). These Lipid A modifications also affect other membrane biophysical properties, altering LPS fluidity, packing, and interactions with divalent cations (Wu et al. 2013; Rice and Wereszczynski 2018). These properties are key in modulating interactions with antimicrobial agents, enabling AMR acquisition against outer membrane antimicrobials, such as the CAMP polymyxin (Olaitan et al. 2014). Gram-positive bacteria also possess methods for enzymatic modification of lipids to modulate membrane surface charge. For example, they can reduce CAMP susceptibility by increasing production of cationic lysyl-PG, which lessens interactions with cationic antimicrobials by increasing electrostatic repulsion (Willdigg and Helmann 2021). In particular, the MprF system, which catalyses the aminoacyl-tRNA-dependent lysylation of PG to lysyl-PG, also acts as a flippase (Ernst and Peschel 2011), translocating lysl-PG from the inner to the outer leaflet, which reduces the overall negative membrane surface charge and provides protection against CAMPs (Andrä et al. 2011; Mishra et al. 2011; Kumariya et al. 2015; Ernst et al. 2018; Sabat et al. 2018; Ernst and Peschel 2019). In addition to lysl-PG formation, resistance phenotypes can also exhibit the aminoacyl-tRNA dependent formation of cationic arginyl-PG or zwitterionic alanyl-PG, highlighting the range of chemical modification of membrane lipids (Sohlenkamp et al. 2007; Klein et al. 2009; Roy and Ibba 2009). Gram-positive bacteria also possess a range of other charge modification systems, including the Dlt system which modifies poly-anionic lipoteichoic acids (LTAs) through the transfer a *D-*alanine moiety, reducing the overall membrane surface charge and providing protection against CAMPs (McBride and Sonenshein 2011; Sabat et al. 2018).

### Acyl tail remodelling

Lipid acyl tail remodelling (Fig. 1E, Table 1) impacts membrane thickness and membrane fluidity and has been implicated in resistance to antimicrobials (Aricha et al. 2004; Maria-Neto et al. 2015; Kumariya et al. 2015). Collectively, bacterial acyl tail remodelling includes changes in unsaturated fatty acid content and speciation, acyl tail length and the relative proportions of acyl tails that incorporate cyclopropane or branched lipid tails (Sohlenkamp and Geiger 2015; López-Lara and Geiger 2017). Experimental studies of lipid bilayers indicate longer, more saturated, and minimally branched lipid tails promote thick, ordered, compact membranes with slowly diffusing lipids (Filippov et al. 2007; Kučerka et al. 2011; Poger et al. 2014; Levental et al. 2016; Marquardt et al. 2016). This is important to consider as acyl tail induced changes to membrane fluidity and ordering have been implicated in resistance to AMPs. For example, increased unsaturation can inhibit the assembly of daptomycin oligomers, modulating daptomycin pore formation (Taylor et al. 2017; Beriashvili et al. 2018). Consequently, increased unsaturated fatty acid content has been observed in resistance to daptomycin and other lipophilic peptide antimicrobials, including colistin (Saito et al. 2014; Harp et al. 2016; Tao et al. 2021). Increases in unsaturation and concomitant decreases in cyclopropane containing lipids that provide resistance to apidaecin 1b or naringenin have been shown to alter membrane fluidity (Wang et al. 2018; Schmidt et al. 2018). In addition to changing membrane biophysical properties, acyl tail remodelling can occur as a secondary effect from changes to enzymatic pathways that provide resistance. For example, mutations in pgsA, resulting in decreased PG content, can also result in upstream accumulation of fatty acids and subsequent remodelling of bacterial lipid fatty acid profiles (Hines et al. 2017). The acyl tail profile of some pathogens is also influenced by the availability of exogenous fatty acids, such as host-derived long-chain polyunsaturated fatty acids which have antimicrobial properties (Yao and Rock 2015; Churchward et al. 2018; Adams et al. 2021; Kengmo Tchoupa et al. 2021). Acyl-tail remodelling induced by exogenous fatty acids has been associated with reduced fitness and increased antimicrobial susceptibility in several pathogens (Kengmo Tchoupa et al. 2021), raising the possibility of combining membrane-active antimicrobial therapy with exogenous fatty acids for increased efficacy.

### Lipid domains and curvature

Some membrane-active antimicrobials target or have their activity modulated by segregated membrane lipid domains (Table 1). The formation of segregated lipid domains is influenced by bacterial membrane composition and biophysical properties, showing dependencies on headgroup content, intrinsic lipid curvature and relative tail order (Epand and Epand 2009b). In bacteria, curvature driven segregation of CL and PE domains at the cell poles is thought to play key roles in polar protein localisation (Matsumoto et al. 2006; Mileykovskaya and Dowhan 2009; Renner and Weibel 2011; Beltrán-Heredia et al. 2019). Some amphiphilic aminoglycosides target CL rich domains, including those at the poles, resulting in domain disassembly and disrupting overall cellular function (El Khoury et al. 2017; Swain et al. 2018). Interactions of antimicrobials with CL rich domains are proposed to be driven by a combination of high negative charge density, high intrinsic curvature and the unique domain segregation preferences of CL (El Khoury et al. 2017; Swain et al. 2018). Increases in glycolipid content are also observed in some antimicrobial resistant strains (Hines et al. 2017). In experimental studies of model lipid bilayers, increases in glycolipid content results in the formation of ordered domains with high structural integrity (Levental et al. 2020) which may play a role in modulating antimicrobial mediated membrane disruption. In addition to targeting pre-existing membrane domains, the mechanism of action of several antimicrobials involves either the induction of altered lateral lipid domains to destabilise the membrane, or the formation of domains that disrupt protein localisation and function (Epand and Epand 2009a; Scheinpflug et al. 2017; Su et al. 2020).

## Lipid transport systems—a further menace

The transport of lipids between membranes or across membrane leaflets can mediate changes in membrane structure and biophysical properties that limit the interaction of antimicrobials with the membrane, affecting antimicrobial susceptibility (Fig. 1F) (May and Grabowicz 2018; Bogdanov et al. 2020; Paulowski et al. 2020). In the outer membrane of Gram-negative bacteria, a number of regulatory systems help maintain the highly asymmetric distribution of lipopolysaccharides between the inner and outer leaflet, required for both membrane integrity and resistance to antimicrobials (May and Grabowicz 2018). In the Gram-negative outer membrane, the phospholipase PldA plays an integral role in membrane homeostasis and membrane asymmetry, by directly degrading phospholipids mis-localised to the outer leaflet of the outer membrane (May and Silhavy 2018). Between the Gram-negative inner and outer membranes, the Mla and Lpt transport systems are integral to the transport of lipids and lipopolysaccharides, respectively (Malinverni and Silhavy 2009; Sperandeo et al. 2017). These systems are critical for bacterial fitness and antimicrobial resistance, and mutations in these systems are present in AMP resistant strains (Lewis et al. 2009; Spohn et al. 2019). Other membrane homeostasis pathways involve the Lipid A palmitoyltransferase, PagP, which transfers a palmitoyl chain from mis-localised lipids to Lipid A (Guo et al. 1998; Bishop 2005; Boll et al. 2015). As previously noted, the MprF system is key in regulating lysyl-PG translocation between the inner and outer leaflets of the Gram-positive membrane (Ernst and Peschel 2011). All of these mechanisms function to preserve the lipid-dependent biophysical properties of bacterial membranes that allow them to function as a key protective barrier. As such, these protein systems are possible targets for future antimicrobial development.

## Membrane efflux pumps—the bacterial empire strikes back

Another pertinent mechanism of membrane-mediated AMR is the active efflux of compounds from the bacterial cell membrane by membrane-embedded transport proteins that act as drug efflux pumps (Fig. 1G) (Henderson et al. 2021). Bacteria can upregulate the expression of drug efflux pumps to efflux a range of substrates, including antimicrobial peptides, lipids, and other antimicrobials (Du et al. 2018). Drug efflux pumps are not only involved in the active extrusion of lipid substrates, but their functioning is also linked to their membrane lipid microenvironment (Fig. 1G inset) (Corradi et al. 2019; Stieger et al. 2021). There are seven key drug efflux superfamilies present in bacteria: the ABC (ATP binding cassette), RND (resistance-nodulation-cell-division), MFS (major facilitator superfamily), MATE (multidrug and toxic compound extrusion), DMT (drug/metabolite transporter), PACE (proteobacterial antimicrobial compound efflux), and AbgT (p-aminobenzoyl-glutamate transporter) families (Henderson et al. 2021). While many of these transporters are found in both Gram-positive and Gram-negative bacteria, Gram-negative bacteria also contain tripartite efflux systems, in which a RND, ABC or MFS inner membrane transporter is coupled to an outer membrane porin or channel via a periplasmic coupling protein. These tripartite efflux systems are a powerful first-defence mechanism for efficient antimicrobial efflux across both the inner and outer membrane of Gram-negative bacteria (Henderson et al. 2021).

Although antimicrobials have only been in widespread use since the 1940s, the ubiquitous distribution of drug efflux pumps across bacterial species indicates they have underlying physiological roles in addition to antimicrobial efflux. Many of these efflux pumps are also associated with membrane homeostasis, or act as virulence factors by facilitating bacterial colonization through the efflux of xenobiotics such as host-derived hormones, signaling molecules or fatty acids (Henderson et al. 2021) (Table 2). In *Escherichia coli*, the ABC multidrug efflux pump MsbA plays a critical role in the transport of Lipid A and LPS for assembly in the cell envelope (Mi et al. 2017; Voss and Stephen Trent 2018). MsbA also effluxes the antimicrobial ethidium (Singh et al. 2016), and the drug daunorubicin (Siarheyeva and Sharom 2009). Investigations into MsbA-substrate interactions show daunorubicin binding decreases the binding affinity of Lipid A to MsbA (Siarheyeva and Sharom 2009). This suggests that in the presence of drugs and antimicrobials, the affinity of MsbA for its natural lipid substrates is decreased, instead facilitating the preferential efflux of antimicrobials.

**Table 2:** Summary of efflux pump lipid interactions

Efflux pump	Bacterial species	Lipid interactions	References
MsbA	*Escherichia coli*	Lipopolysaccharide and Lipid A transport	(Siarheyeva and Sharom 2009; Singh et al. 2016; Mi et al. 2017)
TmrAB	*Thermus thermophilus*	Modulation by phosphatidylglycerol	(Bechara et al. 2015)
MtrD	*Neisseria gonorrhoeae, Neisseria meningitidis*	Membrane-active antimicrobial, bile salt, steroid, and fatty acid efflux	(Shafer et al. 1998; Tzeng et al. 2005; Warner et al. 2008)
AcrB	*Klebsiella pneumoniae*	Membrane-active antimicrobial, bile salt, steroid, and fatty acid efflux	(Padilla et al. 2010)
AdeB	*Acinetobacter baumannii*	Modulation by host-derived polyunsaturated lipids	(Zang et al. 2021)
AcrB	*Escherichia coli*	Modulation by cardiolipin	(Du et al. 2020)
LmrP	*Lactococcus lactis*	Modulation by phosphatidylethanolamine and cardiolipin	(Martens et al. 2016)
pfMATE	*Pyrococcus furiosus*	Modulation of protein conformation by lipid environment	(Zakrzewska et al. 2019; Jagessar et al. 2020)
EmrE	*Escherichia coli*	Modulation of protein assembly by lipid environment	(Nathoo et al. 2013; Dutta et al. 2014)

^a^Key references from this review are presented, and entries are ordered as they appear in text

### ABC efflux pumps

There is mounting evidence that the function and modulation of many membrane proteins, including drug efflux pumps, is influenced by the local membrane environment and its biophysical properties (Corradi et al. 2019) (Table 2). A key example of this is the archetypal eukaryotic multidrug efflux pump, P-glycoprotein (P-gp, ABCB1), a member of the ABC transporter superfamily. P-gp function is increased in the presence of membrane cholesterol and modulated by the overall ordering and phase of the membrane (Rothnie et al. 2001; Sharom 2014). This previous work on eukaryotic drug transporters has provided evidence for a conserved functional mechanism of lipid modulation of bacterial ABC multidrug efflux pumps (Neumann et al. 2017). For example, mass spectrometry analysis of the ABC multidrug efflux pump TmrAB, from *Thermus thermophilus,* has shown that the integrity of the lipid annulus and the high-affinity binding of annular PG lipids to TmrAB is essential for both the structural integrity of the transporter and its ability to hydrolyse ATP to power antimicrobial efflux (Bechara et al. 2015).

### RND efflux pumps

Bacterial RND multidrug efflux pumps actively efflux a range of membrane associated substrates (Table 2). For example, in the human pathogens *Neisseria gonorrhoeae* and *Neisseria meningitidis,* the RND efflux pump, MtrD, is essential for both bacterial virulence and the efflux of a range of host-derived, lipid-based antimicrobials including bile salts, progesterone and fatty acids, as well as other pharmaceutical antimicrobials (Shafer et al. 1998; Warner et al. 2008), and the structurally diverse antimicrobial peptides LL-37, protegrin-1 and polymyxin B (Tzeng et al. 2005). Recent simulation studies of MtrD have demonstrated that the binding of the antimicrobial hormone progesterone to MtrD induces allosteric couplings that govern efflux (Fairweather et al. 2021), and that these allosteric couplings can be deregulated by mutations that impact substrate uptake and the orientation of MtrD within the membrane (Chitsaz et al. 2021). Additionally, the *Klebsiella pneumoniae* AcrAB RND multidrug efflux pump is implicated in resistance to a number of antimicrobial peptides, including polymyxin B (Padilla et al. 2010).

The function of RND efflux pumps is also modulated by their membrane lipid environment. Recent studies linking the membrane composition and AMR in *Acinetobacter baumannii* have revealed an important link between membrane homeostasis, antimicrobial susceptibility and RND efflux pump function (Jiang et al. 2019b; Zang et al. 2021). Modifications of the membrane lipid composition due to incorporation of host-derived polyunsaturated fatty acids in bacterial lipid synthesis gave decreased resistance to antimicrobials. Analysis of *A. baumannii* efflux systems indicated that AdeABC-mediated efflux was impacted by this change in membrane lipid composition, due to a reduction in membrane ordering that abrogated the integrity of the protein–protein interface between AdeB subunits, disrupting AdeB functioning (Zang et al. 2021). Lipid modulation of RND transporter functioning has also been demonstrated for the *E. coli* AcrB RND transporter, whose interaction with a small regulatory protein AcrZ was modulated by the presence of CL, thus resulting in increased sensitivity to chloramphenicol in the absence of CL (Du et al. 2020).

### Other efflux pump families

Lipid modulation of other efflux pump families has also been noted (Table 2). The MFS efflux pump LmrP from *Lactococcus lactis* undergoes proton-dependent conformational transitions during antimicrobial efflux. These conformational changes are sensitive to the presence of PE lipids and CL; however, differences in acyl chain length do not impact conformational switching (Martens et al. 2016). Likewise, the *Pyrococcus furiosus* ion-coupled MATE antimicrobial antiporter, pfMATE, is also strictly lipid dependent: the protein is only functional when reconstituted in a lipid environment (not in detergent), highlighting functional dependence on the presence of lipids (Zakrzewska et al. 2019; Jagessar et al. 2020). Additionally, the assembly of an *E. coli* ion-coupled small multidrug resistance (SMR) transporter EmrE, responsible for resistance to aromatic cationic compounds, is dependent on its lipid environment (Schuldiner 2009; Nathoo et al. 2013; Dutta et al. 2014).

Collectively, these examples highlight the growing body of evidence demonstrating that the lipid environment is integral to the function and modulation of many multidrug efflux pumps that are critical to the development of AMR. The importance of this must be considered to gain a holistic understanding of the impact of the membrane in modulating multidrug resistance.

## Understanding the role of lipid-mediated resistance for antimicrobial development—a new hope

**Fig. 2:**
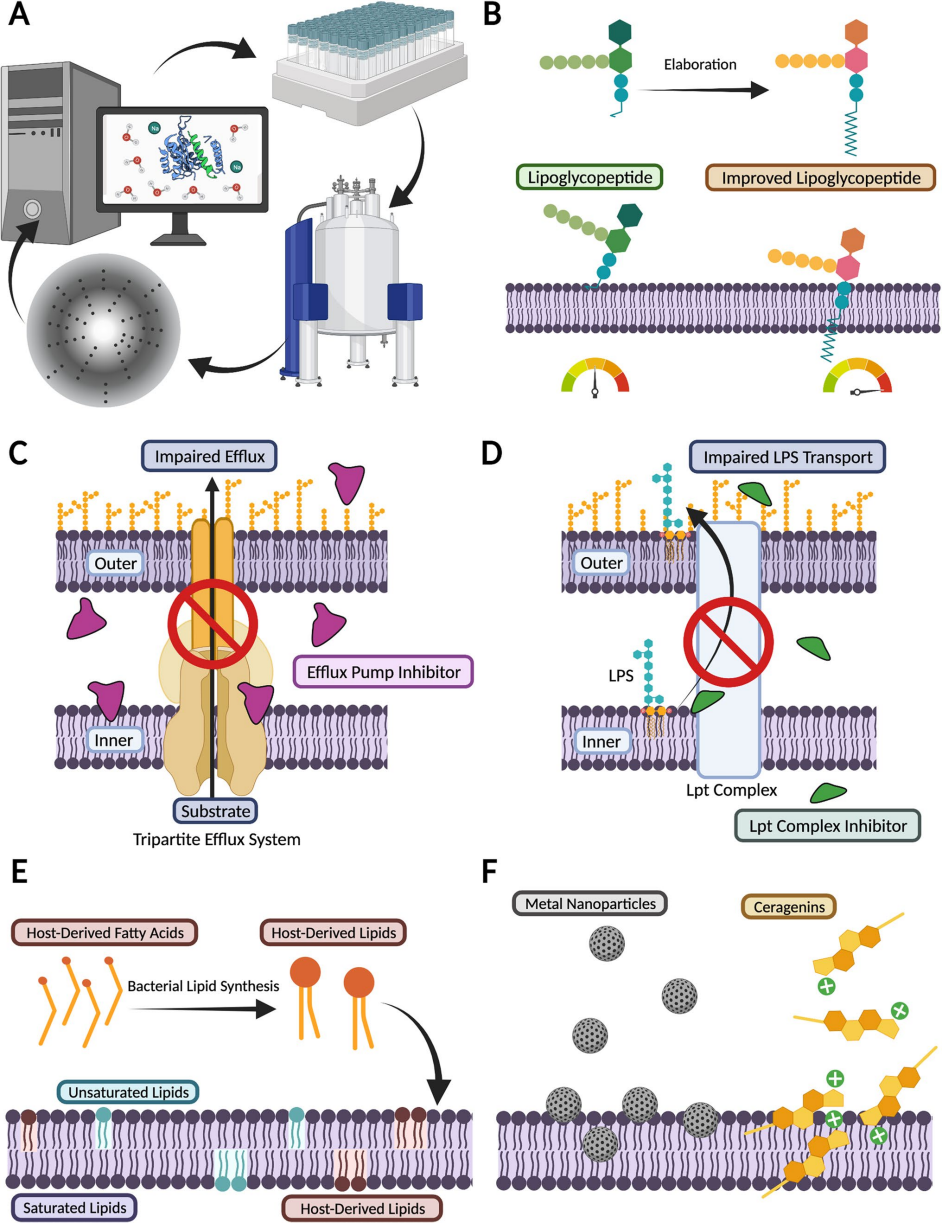
Guiding the development of novel membrane-active antimicrobials. (A) Iterative cycles of in vivo and in vitro experiments, combined with experimental biophysical techniques, such as NMR and X-ray crystallography, and computational approaches allows for the streamlined and rational design of novel antimicrobials. (B) Modified AMPs, such as lipoglycopeptides with added lipophilic groups allow for enhanced membrane interactions. (C) The inhibition of drug efflux systems and (D) lipid transport systems are emerging membrane-associated drug targets. (E) Host-derived fatty acids can have antimicrobial activity against membranes via changes to membrane biophysical properties. (F) Non-peptide cationic antimicrobials, such as metal nanoparticles and steroid-based antimicrobials can interact with negatively charged bacterial membranes to exert their antimicrobial properties. Figure created with BioRender.com

The development of membrane-active antimicrobials is a multi-faceted area of research. Extensive links between the efficacy of membrane-active antimicrobials and lipid-mediated AMR means that successful antimicrobial development must be founded on a detailed understanding of membrane dynamics, membrane biophysical properties and the functional mechanisms of transporters involved in AMR. This is best achieved using a combination of experimental techniques and computational approaches (Fig. 2A) (Fjell et al. 2012; Lopes et al. 2017; Li et al. 2017). Although, experimental methods, such as nuclear magnetic resonance, X-ray crystallography, circular dichroism and cryo-EM provide valuable structural information about bacterial cell membranes and membrane proteins, they are often limited by either temporal or spatial resolution. In silico approaches, such as molecular dynamics simulations, allow for high resolution characterisation of the functional dynamics and interactions between antimicrobials and their membrane targets (Berglund et al. 2015; Ulmschneider and Ulmschneider 2018; Palmer et al. 2021). Thus, iterative combinations of molecular simulations and experimental methodologies will allow for the streamlined, innovative, and rational design of novel antimicrobials (Li et al. 2015; Chen et al. 2019).

### Antimicrobial peptides

The continued exploration into naturally occurring antimicrobial peptides (AMPs) from the innate host immune response has created a vast natural library of membrane-active compounds (Ashby et al. 2017). Whilst AMPs generally carry a net positive charge, their large variations in size (between ~ 9 and 100 amino acids) and structural diversity (they can be linear or cyclical and adopt α-helical, β-sheet or mixed structures) (Shai 2002) makes AMPs and AMP-based structural modifications a promising future direction for expanding the current range of antimicrobials. Additionally, AMPs can act as conjunct therapeutics to restore antimicrobial sensitivity in resistant bacteria (Lin et al. 2015; Pizzolato-Cezar et al. 2019). Growing computational and experimental insight into the interactions between AMPs and the membrane has already allowed for the development of modified AMPs with improved action (Fig. 2B). For example, lipoglycopeptides such as telavancin, oritavancin and dalbavancin are derivatives of earlier generation glycopeptides, with the addition of lipophilic alkyl and aryl groups to the sugar subunit. These lipophilic moieties act as membrane anchoring groups, enhancing interactions with the hydrophobic lipid tails, resulting in membrane permeabilization and the loss of membrane integrity (Blaskovich et al. 2018).

### Efflux pump inhibitors

Membrane-active peptides are also inhibitors of some multidrug efflux pumps, opening avenues for the inhibition of efflux pump systems via the co-administration of known or engineered peptides (Fig. 2C). This strategy has been effective against the RND efflux pump AcrB in *E. coli* (Jesin et al. 2020), and against an SMR efflux protein of *Pseudomonas aeruginosa,* PAsmr, inhibiting antimicrobial efflux (Mitchell et al. 2019). These and other efflux pump inhibitors (EPIs), including PAβN, pyridopyrimidines, quinoline derivatives, arylpiperidines and arylpiperazines have been identified as having the potential to abrogate resistance in a number of problematic pathogens (Kabra et al. 2019). Continued exploration of the peptide EPI sequence space, coupled with improved knowledge of the functional mechanisms of drug efflux pumps, and their relationship with membrane composition and membrane biophysical properties will better enable the development of highly efficacious and specific membrane-active AMPs for resistant bacterial pathogens.

### Targeting lipid synthesis and transport

Another area for the development of membrane-active agents includes the targeting of proteins involved in lipid synthesis and membrane homeostasis (Fig. 2D). Potential protein targets include the condensing enzymes of the fatty acid biosynthesis cycle (FabH, FabB, FabF); the *sn*-glycerol-3-phosphate acyltransferase (PlsB) involved in linking fatty acids to the phospholipid glycerol-3-phosphate backbone; or members of the Lpt complex involved in transport of LPS to the extracellular leaflet of the membrane (Heath et al. 2001; Heath and Rock 2004; Choi and Lee 2019; Walker and Black 2021). For example, the outer membrane assembly inhibitor, murepavadin, which is based on the protegrin-1 AMP, is a likely inhibitor of members of the Lpt complex (Lehman and Grabowicz 2019). Further exploration of lipid synthesis and transport inhibitors is needed to better target this under-exploited avenue for membrane-active antimicrobial development.

### Host-derived fatty acids

Host-derived fatty-acids, including lauric and sapienic acid, can also act as membrane-active antimicrobials (Fig. 2E) (Thormar and Hilmarsson 2007; Fischer 2020). However, the mechanism of action of these fatty acids is highly varied, and includes the disruption of oxidative phosphorylation via binding to membrane embedded proteins, alteration of membrane biophysical properties and permeability, and the inhibition of enzymes involved in fatty acid synthesis pathways (Desbois and Smith 2010). Detailed computational and experimental investigation of the mechanisms by which host-derived antimicrobial fatty-acids disrupt the bacterial membrane may enable the renewal of existing therapeutics through co-administration of host-derived membrane-active fatty acids.

### Non-peptide cationic antimicrobials

Due to the high concentration of negatively charged lipids in bacterial membranes, there is increasing interest in the development of non-peptide cationic antimicrobial agents that use a combination of electrostatics and lipophilicity to bind to and disrupt bacterial membranes (Fig. 2F). One such application is the design of membrane-disrupting metal nanoparticles and metal ion clusters which increase membrane permeability and promote oxidation of bacterial membrane lipids (Godoy-Gallardo et al. 2021). Cationic steroid antimicrobials such as ceragenins also utilise this mechanism. Ceragenins are synthetic sterol-based compounds decorated with amino acids or other chemical groups to aid lipid partitioning. These cationic compounds cause membrane permeabilization and depolarization. Importantly, the efficacy of ceragenins is impacted by membrane PE and anionic lipid content (Epand et al. 2007, 2010). As with AMPs, understanding of the biophysical mechanisms by which non-peptide cationic antimicrobials disrupt the bacterial membrane will aid in their development and enhance their effectiveness.

### Computational approaches

Molecular dynamics simulations are poised to play an essential role in elucidating the membrane and transporter dynamics critical to lipid-based AMR. Importantly, bacterial lipid composition can now be examined using high resolution mass-spectrometry techniques allowing unprecedented insight into the lipid composition of bacterial membranes and responses to antimicrobials (Rustam and Reid 2018; Appala et al. 2020). When combined with recent advances in cryo-EM structural elucidation, structure-based drug design, as well as improved computational hardware and algorithms, these advances allow for the development of detailed molecular models that can reveal the mechanisms that underpin lipid-mediated AMR in bacteria. Molecular simulation of bacterial membranes, and biological membranes in general, has advanced significantly over the past 20 years, with larger, more complex and more realistic simulations becoming feasible (Marrink et al. 2019; Wilson et al. 2020b; Im and Khalid 2020). Advances in forcefields, particularly the CHARMM atomistic forcefield and MARTINI coarse-grained forcefield (Marrink et al. 2004, 2007; Wu et al. 2014; Lee et al. 2019a), coupled with increasing hardware capabilities and improvements in simulation tooling, has enabled simulations of membranes approaching realistic chemical diversity in constituent protein and lipid components (Ingólfsson et al. 2014, 2017; Reddy et al. 2015; Wilson et al. 2020a, 2021).

For example, large scale coarse-grained simulations have identified the preferential interaction of some AMPs with ordered domains in phase separated membranes (Su et al. 2020). Molecular dynamics simulations have also been used to show the preference of AMPs to bind in regions of high membrane curvature, as well as the curvature-dependence of AMP secondary structure (Chen and Mark 2011). Simulation work on the mechanisms governing lipid modification induced reduction in AMP binding has examined the role of lysl-PG in AMP surface binding and membrane disruption in a model bacterial membrane (Simcock et al. 2021). Molecular insights into transporter regulation by lipid components and membrane biophysical properties derived from simulations is gaining momentum (Corradi et al. 2018, 2019; Corey et al. 2021) and has recently been applied to AdeB and AdeJ transporter regulation in *A. baumannii* (Zang et al. 2021)*.* Advanced simulations of Gram-negative membranes now contain the inner and outer membrane, LPS and peptidoglycan layers, and multiple copies of inner and outer membrane proteins, enabling even more detailed insights into the molecular mechanisms of lipid-based resistance (Im and Khalid 2020).

## Conclusion

Lipid-mediated antimicrobial resistance is a multi-faceted phenomenon with inter-dependencies arising not only from the lipid membrane composition, but also from bacterial metabolic pathways, sequestration of lipids from the host environment, enzymatic modifications of lipid targets and the activity of drug efflux pumps. These interact synergistically to alter antimicrobial targets, decrease the specificity of membrane-active antimicrobials and actively efflux antimicrobial agents, increasing the inherent AMR of bacteria. As a result of rapidly escalating rates of AMR, there is continued interest in the development of membrane-active antimicrobials that may restore efficacy of now redundant antimicrobials or present new avenues for antimicrobial therapy. When combined with the improvements in the resolution of experimental methods to determine membrane lipid composition, membrane protein structural elucidation and advances in molecular simulation, the stage is set to develop more effective approaches to overcome lipid-mediated antimicrobial resistance.

## Data Availability

Not applicable.
